# Supplementary material for: The healthy context paradox of bullying victimization and academic adjustment among Chinese adolescents: A moderated mediation model
Source: PLoS One. 2023 Aug 18;18(8):e0290452. doi: 10.1371/journal.pone.0290452 (PMC10437784; doi:10.1371/journal.pone.0290452)
Supplement: Attachment — Submitted filename: renamed_0127e.docx [file pone.0290452.s002.docx]

**Dear Editor and Reviewers:**

Thank you for your letter and for the reviewers’ comments concerning our manuscript entitled “The Healthy Context Paradox of Adolescent Bullying Victimization and Academic Adjustment: A Moderated Mediation Model” [PONE-D-23-06364]. We are pleased to know that our work was rated as potentially acceptable for publication, subject to adequate revision. These comments are all valuable and very helpful for revising and refining our paper, as well as providing important guidance for our research. Based on your suggestion and request, we have carefully studied these comments and made corrections, which we hope will meet with your approval. We would also like to thank you for allowing us to resubmit a revised version of the manuscript. Revised sections are marked in red in the paper. Responses to the reviewer’s comments are as flowing:

**Changes regarding financial disclosure:**

The updated statement on financial disclosure is included in the cover letter, as detailed in the section marked in red.

**Response to Journal Requirements:**

1. Please ensure that your manuscript meets PLOS ONE's style requirements, including those for file naming.

**The author’s answer:** In the revised version, we carefully examined the manuscript and made changes to the parts that did not meet the norms. First, for different levels of titles, we changed the format accordingly. **See lines 13, 26, 41, 68, 95, 118, 139, 140, 152, 153, 159, 166, 170, 178, 184, 185, 190, 200, 235, 296, 344, 354.** Second, we added the title to the manuscript and made sure it appeared directly after the paragraph in which they were first cited. **See lines 212, 231-234.** Third, we checked and revised the format of all references to ensure they were consistent and met the requirements of the journal. Finally, we have included the captions of the supporting information files at the end of the manuscript. **See lines 521-522.**

2. Please provide additional details regarding participant consent. In the ethics statement in the Methods and online submission information, please ensure that you have specified what type you obtained (for instance, written or verbal, and if verbal, how it was documented and witnessed). If your study included minors, state whether you obtained consent from parents or guardians. If the need for consent was waived by the ethics committee, please include this information.

**The author’s answer:** Our study was approved by the Research Ethics Committee of the Psychology, College of Education and Sports Sciences, Yangtze University. During the study, we always maintain the important principles of voluntary participation and informed consent. Since the participants in the study were minors, we were informed in advance in order to obtain the consent of their parents or guardians. During the study, their informed consent was reflected by the return of the questionnaire. If either parent or the student does not agree to participate in this survey, the student does not be required to complete the questionnaire if he or she verbally states so. In the participants section, the specific details of obtaining informed consent have been added accordingly. **See lines 144-147**.

3. We note that the grant information you provided in the ‘Funding Information’ and ‘Financial Disclosure’ sections do not match.

When you resubmit, please ensure that you provide the correct grant numbers for the awards you received for your study in the ‘Funding Information’ section.

**The author’s answer:** Thanks for pointing this out. We submitted again with the correct version.

4. We note that you have provided funding information that is not currently declared in your Funding Statement. However, funding information should not appear in the Acknowledgments section or other areas of your manuscript. We will only publish funding information present in the Funding Statement section of the online submission form. Please remove any funding-related text from the manuscript and let us know how you would like to update your Funding Statement. Currently, your Funding Statement reads as follows: The funders had no role in study design, data collection and analysis, decision to publish, or preparation of the manuscript. Please include your amended statements within your cover letter; we will change the online submission form on your behalf.

**The author’s answer:** We removed this information from the resubmitted manuscript. Our Funding Statement reads as follows:

Xiong Gan received fund. This research was supported by Youth project of Science and Technology Research Plan of Department of Education of Hubei Province in 2020 (Q20201306), the Social Science Fund Project of Yangtze University in 2022 (2022csz03), the Faculty Scientific Fund Project of the College of Education and Sports Sciences of Yangtze University in 2022 (2022JTB01), and the key projects of education science plan of Hubei Province in 2022: Study on the influencing factors and intervention mechanism of non-suicidal self-injurious behaviors in adolescents (2022GA030).

**Response to Reviewer #1:**

I am pleased to receive the invitation to review this paper entitled " The Healthy Context Paradox of Adolescent Bullying Victimization and Academic Adjustment: A Moderated Mediation Model", which explores the relationship between bullying victimization and academic adjustment, as well as the mediating effects of subjective well-being and the moderating role of classroom-level victimization. My comments are as follows:

(1) It would help if the author provides more theoretical and empirical base for the debate of “Healthy Context Paradox”.

**The author’s answer:** We have elaborated more on the original manuscript. **See lines** **46-48, 52-55, 59-61, 81-87.**

(2) The authors should give more detailed information about their random cluster sampling process.

**The author’s answer:** Thanks for your suggestion. We have described this in more detail. **See lines140-142.**

(3) The measures used in the study suffer from several drawbacks. The most important one is that it is unclear whether the classroom-level victimization variable is a class level measure or an individual level measure. It must be clarified at which level it has been measured and included in the model, and the modeling must adjust accordingly. In my view, the study could benefit from re-analysis using more appropriate and advanced statistical methods. The authors could have considered using multi-level models, also known as hierarchical linear models, to account for the potential issue of biased estimates or standard errors that can arise from traditional regression models.

**The author’s answer:** Thank you very much for your review and valuable suggestions on our research measures. The measurement method you mentioned has some drawbacks, we fully agree with your point of view and have made corresponding explanations in the revised draft. **See lines 168-169, 337-340.** In our preliminary analysis, we attempted to use hierarchical linear models to construct model and analyze data. However, we found that the Intra-Class Correlation (ICC) indicator did not meet requirements, indicating lower than expected within-group correlation which affected our interpretation and modeling of victim variables at the classroom level. In addition, according to Peugh (2010), when the design effect of the sample is greater than 2, it is necessary to analyze the data using a multilevel model to avoid statistical bias. For some reason, the design effect for one of our variables did not reach 2, so on balance we did not use a multilevel linear model. It is possible that the data structure or the sample size caused these problems, so we recognize that this is a limitation of our statistical approach, and in the future research, we will consider collecting larger samples and ensuring good data structure to construct more appropriate models.

Peugh, J. L. (2010). A practical guide to multilevel modeling. *Journal of School Psychology*, 48(1), 85–112. <https://doi.org/10.1016/j.jsp.2009.09.002>

(4) Bootstrap analysis could be helpful to test the mediating effect.

**The author’s answer:** Thank you for your suggestion. We have used bootstrap analysis in the process of analyzing the mediation effect. It may have been due to our oversight that we did not explicitly state it in the manuscript. We have now improved this part. **See lines 206-211.**

(5) Please give more specific practical implications both on policy level and management level.

**The author’s answer:** Thanks for your advice. We give additional and specific practical implications at both levels. **See lines 314-319, 322-325.**

(6) In addition, there are some grammar errors in your manuscript. Please check it carefully.

**The author’s answer:** Thanks for your careful review, we have checked and corrected it.

**Response to Reviewer #2:**

Thank you for the opportunity to review the manuscript. Certainly, the topic is interesting, relevant and fits the contents of the magazine. I believe it has good potential and great practical relevance in the field of education.

1. The origin of the students should be included in the title.

**The author’s answer:** Thank you for your advice. We have made a change to this. **See lines 1-2.**

2. The abstract defines conceptually different parts of the study and summarizing the content and objectives of the work, contains the required number of words and at least five keywords. Keywords refer to the most important variables or topics of the study.

**The author’s answer:** Thanks for your suggestion. We refined the abstract and keywords. **See lines 14-25.**

3. The justification explained in the manuscript is shallow, some references should be updated, and secondary citations are used.

**The author’s answer:** We provide additional explanations of the healthy environment paradox and update some of the references. **See lines 46-48, 52-55, 59-61, 81-87.**

4. Occasionally, the writing is somewhat confusing (the information should be better organized, some ideas should be lightened, and the discourse should be coherent).

**The author’s answer:** Thank you for your suggestions, we have scrutinized our manuscript and made some changes.

5. The sample selection procedure should be better explained.

**The author’s answer:** We made further clarifications. **See lines 140-142, 144-147.**

6. The research hypotheses should be explained after stating the objectives.

**The author’s answer:** We have explained accordingly in the research hypotheses section. **See lines 125-127, 130-136.**

7. You should better explain the data analysis section and justify the statistics used. I consider that the practical relevance of the results found should be explained in greater depth, not simply describing them, but explaining their practical scope and justifying them according to previous empirical evidence. Sometimes a literal description of the results is made without a discussion of them.

**The author’s answer:** We have revised the relevant sections to provide a more in-depth analysis and interpretation of the findings. **See lines 193-194, 196-197, 206-211, 244, 301-303.**

8. It would be important to include a section after the limitations that includes the main practical implications, in order to better organize the information of the work.

**The author’s answer:** Thanks for your advice. We have added some information about this. **See lines 298-311, 314-319, 322-325.**

9. I believe that it would also be interesting to include other variables that may be mediating the relationship between the variables.

**The author’s answer:** Thanks for your advice. We have provided some clarification on this. **See lines 309-310.**

10. It would be interesting to explain the following statement in the text (page 19, lines 3-5): "This finding provided the first insight into the mechanisms underlying the healthy context paradox of bullying and academic adjustment, and shed light on how the classroom-level victimization affects the academic adjustment of victimized children”.

**The author’s answer:** Thanks for your review. We have explained this further. **See lines 290-295.**

11. On a formal level, the manuscript complies with the requirements of the Journal and references are written in accordance with the regulations of the Journal. It should indicate the exact location of tables and figures in the manuscript.

**The author’s answer:** Thanks for the heads up. We've added the headings where the figure should be in. **See lines 212, 231-234.**

12. You should revise some English expressions and phrases to make the text more coherent.

**The author’s answer:** Thanks for the mention. We've double-checked the entire article and made changes accordingly.

13. The work is ambitious, and the results confirm most of the hypotheses and the relevance and potential of the work is therefore recognized, but this Reviewer considers that several changes are needed to the manuscript is publishable. In this sense, it should better explain the novelty and relevance of the work considering the previous empirical evidence and should better describe the practical implications, and the principal limitations.

**The author’s answer:** Thank you for taking the time to review our manuscript. We appreciate your valuable feedback and constructive comments. We have carefully considered your suggestions and have made the necessary revisions to address the concerns raised. **See lines 298-311, 314-319, 322-325, 337-340.**

14. It should describe the discussion and conclusions of the work better and, above all, update the manuscript references (most should be from the last 5 years). Finally, I wish the Authors the best in continuing this line of research.

**The author’s answer:** We sincerely appreciate your feedback and suggestions for improving the discussion and conclusions of our work. **See lines 290-295, 345-352.** We have made improvements to optimize the discussion and conclusion sections of our manuscript and replaced outdated references with more current and relevant sources.

In short, our original manuscript is relatively immature. Your suggestions have helped us clarify the variable relationships and illuminate the significance of the study. Also, your suggestions for certain misrepresentations were very timely. Based on your questions, we have carefully examined the entire text and added appropriate additions to the text where it is unclear, in order to improve it. These descriptions may not be what you asked for, but based on your suggestions, we think it is necessary after carefully combing through the article. We are grateful and will pay more attention in our future research. Thank you!

Thanks to the reviewer for their approval and suggestions, which helped us to improve the article, especially the description and explanation of the relationship between the variables. We tried our best to improve the manuscript and made some changes in the manuscript. These changes will not influence the framework of the paper. We appreciate for Editors and Reviewers warm work earnestly and hope that the correction will meet with approval.

Once again, thank you very much for your comments and suggestions.

Yours sincerely,

Xiong Gan

July 12, 2023

Department of Psychology, College of Education and Sports Sciences, Yangtze

University, Hubei, Jingzhou 434023, China

E-mail: 307180052@qq.com
